# Elevated *UMOD* methylation level in peripheral blood is associated with gout risk

**DOI:** 10.1038/s41598-017-11627-w

**Published:** 2017-09-11

**Authors:** Yong Yang, Xiaoying Chen, Haochang Hu, Yuting Jiang, Hang Yu, Jie Dai, Yiyi Mao, Shiwei Duan

**Affiliations:** Medical Genetics Center, School of Medicine, Ningbo University, Ningbo, Zhejiang 315211 China

## Abstract

Uromodulin (*UMOD*) encodes an uromodulin glycoprotein, and its mutation results in uromodulin glycoprotein dysfunction and the occurrence of gout. The aim of our study was to assess whether *UMOD* methylation could predict the risk of gout. A total of 89 sporadic gout cases and 103 age and gender-matched healthy controls were recruited in this study. *UMOD* methylation level was determined by quantitative methylation-specific PCR (qMSP) in peripheral blood, and the percentage of methylated reference (PMR) was described to represent the methylation level. Our results showed that *UMOD* methylation was significantly higher in gout cases than controls (median: 1.45 versus 0.75, *P* < 0.001). The area under curve (AUC) of *UMOD* methylation in gout was 0.764 (*P* = 2.90E-10) with a sensitivity of 65.2% and a specificity of 88.3%. *UMOD* methylation level was shown to be significantly correlated with the serum level of uric acid (UA) (r = −0.208, *P* = 0.035). Besides, the luciferase reporter assay showed that *UMOD* CpG island region was able to upregulate gene expression (fold change = 2, *P* = 0.004). In conclusion, *UMOD* methylation assessment might be used to predict the occurrence of gout.

## Introduction

Gout is one of the oldest described rheumatic diseases. It affects 1–2% of the global population^1^. Gout is a complex disease with much severe comorbidity^2^. There are many risk factors for the development of gout, including hyperuricaemia, dietary factors, alcohol consumption, metabolic syndrome, hypertension, obesity, diuretic use, chronic renal disease, and genetic factors^3, 4^. Although effective treatments were available for gout, drug uptake remained poor. Many patients may experience repeated acute attacks and greatly reduces quality of life^3^. Therefore, the exact pathogenesis of gout is still needed to be explored.

DNA methylation is the most common but crucial way of epigenetic mechanisms^5^. Genes with aberrant DNA methylation contributed to the risk of diseases or disorders such as coronary heart disease^6^, cancer^7^, essential hypertension^8^, leukemia^9^ and type 2 diabetes^10^. However, little research about the association between DNA methylation and the pathogenesis of gout has been reported.

Uromodulin (*UMOD*) is located at the short arm of chromosome 16 and consists of 11 exons^11^. Previous study showed that gout was associated with *UMOD* gene mutations^12^. *UMOD* gene variants were associated with susceptibility to the risk of chronic kidney disease in several genome-wide association studies^13^. Besides, *UMOD* variants were involved in hypertension^14, 15^ and end-stage renal disease^16^. Therefore, we supposed that *UMOD* methylation might play a potential role in the occurrence of gout. In this study, we measured *UMOD* methylation level in peripheral blood to explore its association with gout in Chinese Han male population.

## Materials and Methods

### Sample selection

A total of 89 gout cases and 103 age-matched controls were selected from Ningbo No. 2 Hospital in Zhejiang province of China. All the individuals were Chinese Han males, and the details of their clinical information were shown in Table 1. The mean age of gout patients was 51.52 ± 14.27 years compared with 49.95 ± 12.04 years of the healthy controls.

**Table 1:** The characteristics of cases and controls.

Characteristics	Case (n = 89)	Control (n = 103)	*P* value*
Age (yrs)	51.84 ± 14.09	50.01 ± 12.09	0.393^**b**^
ALT (U/L)	26.00 (19.00, 42.50)	21.00 (17.00, 26.00)	**<0.001** ^a^
AST (U/L)	24.00 (18.50, 31.00)	22.00 (18.00, 27.00)	0.093^**a**^
CRE (mmol/L)	80.52 ± 16.66	77.77 ± 9.79	0.159^**b**^
UA (mmol/L)	423.46 ± 147.48	344.20 ± 67.06	**<0.001** ^**b**^
Glu (mmol/L)	5.30 (4.83, 6.00)	4.96 (4.72, 5.25)	**<0.001** ^**a**^
Cholesterol (mmol/L)	4.94 ± 1.17	4.42 ± 0.68	**<0.001** ^**b**^
HDL (mmol/L)	1.23 ± 0.31	1.50 ± 0.35	**<0.001** ^**b**^
LDL (mmol/L)	2.51 ± 0.98	2.60 ± 0.52	0.427^**b**^
TG (mmol/L)	2.52 ± 1.52	1.17 ± 0.47	**<0.001** ^**b**^
WBC (×10^9^/L)	8.97 ± 3.48	6.42 ± 1.56	**<0.001** ^**b**^

^*^The value in bold indicates statistical significance.

^a^Not conform to normal distribution, nonparametric rank test was applied, and the results were described as median (interquartile range). ^b^Conform to normal distribution, two-sample t-test was applied, and the variables were described as mean ± SD.

ALT: glutamic pyruvic transaminase; AST: glutamic oxalacetic transaminase; CRE: creatinine; UA: uric acid; Glu: blood glucose; HDL: high-density lipoprotein; LDL: low-density lipoprotein; TG: triglyceride; WBC: white blood cell.

The study protocol was approved by the Ethical Committee of Ningbo No. 2 Hospital. All methods were carried out in accordance with relevant guidelines and regulations. Written informed consent forms were obtained from all subjects.

Plasma levels of glutamic pyruvic transaminase (ALT), glutamic oxalacetic transaminase (AST) were determined by the velocity method^17, 18^. The concentrations of creatinine (CRE), uric acid (UA), blood glucose (Glu) and triglyceride (TG) in plasma were determined using the classic enzymatic methods^19–22^. Cholesterol level was measured using automated enzymatic methods^23^. High-density lipoprotein (HDL) cholesterol concentration was measured by enzymatic colorimetric methods with commercially available kits, and low-density lipoprotein (LDL) cholesterol concentration was measured by homogeneous assay^24^. The number of white blood cell (WBC) was measured by a standard blood test^25^.

### DNA methylation analysis

The details of human genomic DNA extraction and concentration determination were as previously described^10^. DNA methylation was modified by EZ DNA Methylation-Gold^TM^ kit (Zymo Research Corporation, Irvine, CA, USA). DNA methylation level was measured by quantitative methylation-specific PCR (qMSP) using the LightCycler^®^ 480 machine (Roche Diagnostics, Mannheim, Germany). To avoid errors that may occur from differences in the loading quantity of the samples, *ACTB* was taken as the internal reference. We used 100% SssI-treated sperm DNA as a positive control^26^, and nuclease-free water as a negative control for each panel. The qMSP was performed in a total volume of 10 µl and contained 5 µl of 2× SYBR Green Master Mix, 0.25 µl primers, 4 µl of ddH_2_O and 0.5 µl DNA. The primers were as follows: *UMOD*, forward 5′-GTTGTTGTTGGCGGAGTA-3′ and reverse 5′-CGACGATAACCTAACCTACG-3′; *ACTB*, forward 5′-TGGTGATGGAGGAGGTTTAGTAAGT-3′ and reverse 5′-AACCAATAAAACCTACTCCTCCCTTAA-3′. PCR amplification procedure included an initial denaturation at 95 °C for 10 min, 45 cycles of denaturation at 95 °C for 20 sec, annealing at 59 °C for 30 sec and extension at 72 °C for 30 sec. A melting curve procedure included 95 °C for 15 sec, 58 °C for 60 sec and 0.11 °C per second up to 95 °C. The amount of methylated DNA (PMR, percentage of methylated reference) at a specific locus was calculated by dividing the *UMOD*:*ACTB* ratio of a sample by the *UMOD*:*ACTB* ratio of SssI-treated human sperm DNA (presumably fully methylated)^26^.

### Luciferase reporter gene assay

The human embryonic kidney 293 T (HEK293T) cell line was cultured as previously described^27^. The fragment of *UMOD* (+7151 bp to +7550 bp), *GCKR* (−173 bp to +227 bp), *COMT* (−386 bp to +14 bp) and *CCL2* (−537 bp to −138 bp) were chemically synthesized and were digested with XhoI and KpnI (New England Biolabs, Ipswich, MA). The target DNA fragment, purified by Cycle Pure Kit (Omega, Norcross, GA, USA), was cloned to pGL3 Basic vector in the presence of DNA Ligation Kit (TaKaRa, Japan). The empty pGL3-Basic vector was used as negative control, and the pGL3-Control vector, (Promega, Madison city, WI, USA) containing an SV40 promoter upstream of the luciferase gene was used as positive control. Cells were prepared in 96-well plates and the details of plasmids transfection were as described previously^28^. After 18–72 h of HEK293T cells transfection, renilla and firefly luciferase activity was measured by SpectraMax 190 (Molecular Devices, Sunnyvale, USA). Luciferase activity was determined with the dual luciferase reporter assay system (Dual-Luciferase^®^ Reporter Assay Systems, Promega, Madison city, WI, USA).

### Statistical analysis

All the statistical analyses were performed by SPSS software version 18.0 (SPSS, Inc., Chicago, IL, USA). Comparisons of the PMR differences between the gout cases and controls were performed by non-parametric test. The correlations between *UMOD* methylation and clinical features were assessed by Spearman test. Receiver operating characteristic (ROC) curves were generated to confirm the diagnostic accuracy of *UMOD*. *P* value less than 0.05 was considered to indicate a statistically significant difference.

## Results

In the current study, only the male samples were selected since gout was predominant in males (a male/female ratio of 4:1)^29, 30^. As shown in Table 1, a total of 11 clinical characteristics were collected from all the individuals. Significantly lower level of HDL was found in the gout cases than controls (mean ± sd: 1.23 ± 0.31 versus 1.50 ± 0.35, *P* < 0.001). Meanwhile, significantly higher levels of ALT, UA, Glu, cholesterol, TG and WBC were found in the gout cases than controls (all *P* ≤ 0.001).

**Figure 1:**
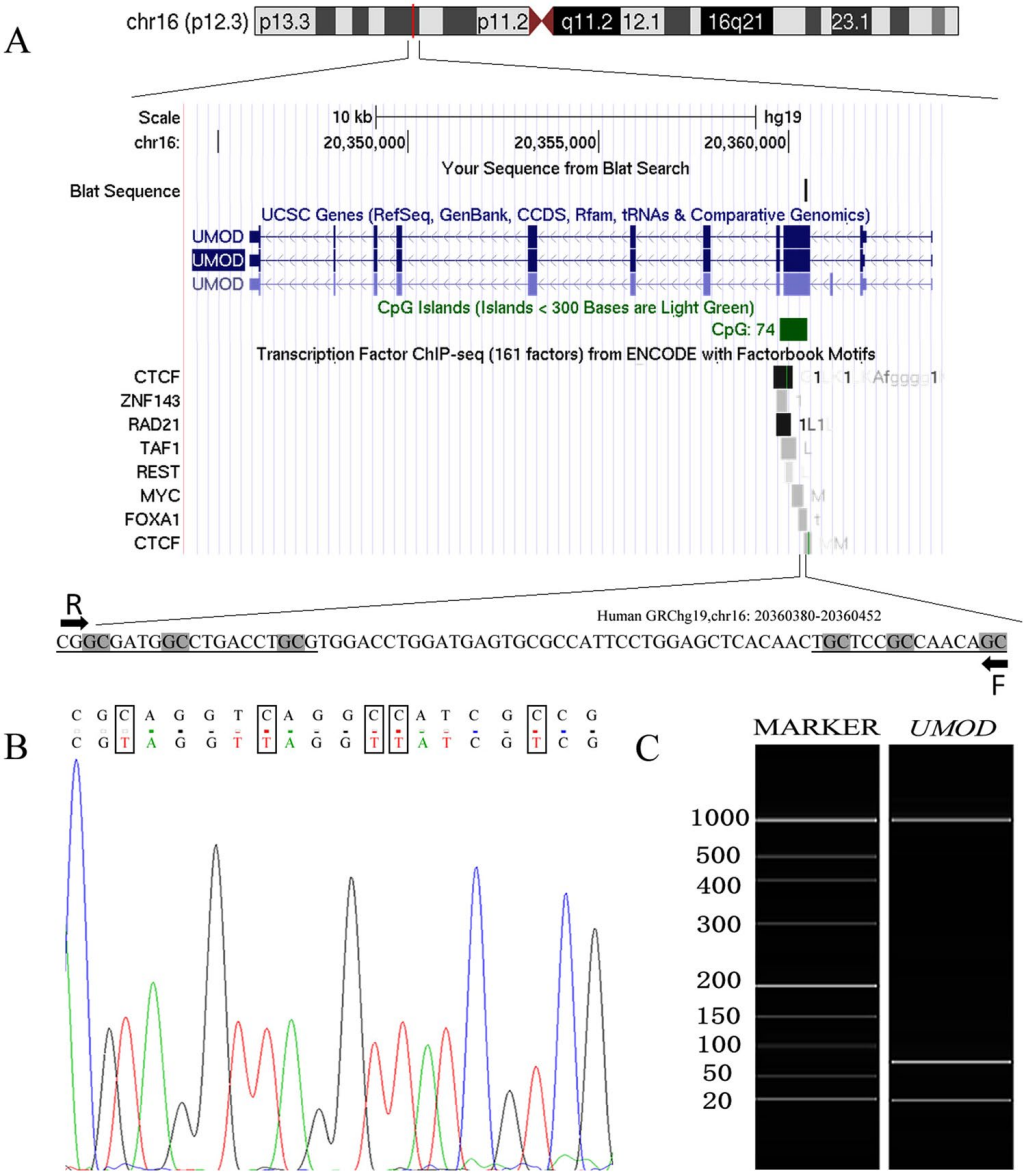
The characteristics of target sequences in *UMOD* gene. Target sequences on *UMOD* gene CpG island region. (**A**) The target sequence is located on the CpG island of *UMOD* gene (location). F stands for forward primer and R stands for reverse primer. (**B**) Sequencing validation of the MSP product. The top row of the sequences represents the original gene sequence, and the second row shows the converted sequence. (**C**) The fragment length of MSP product is 73 bp.

**Figure 2:**
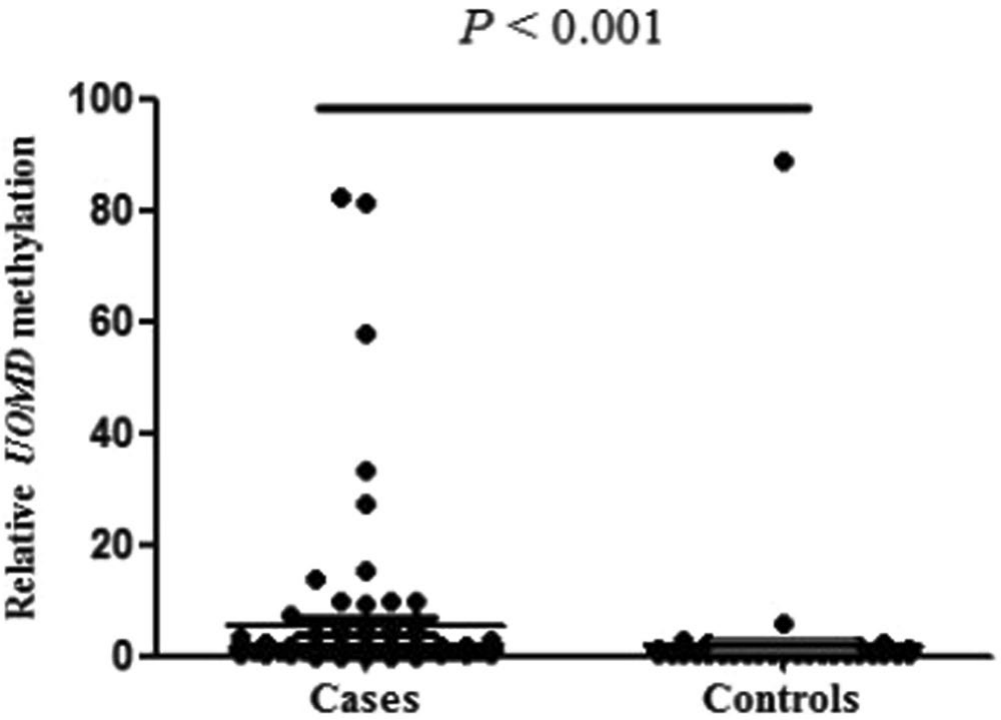
Comparison of relative *UMOD* methylation levels between gout and controls. The levels of *UMOD* methylation are represented by percent of methylated reference (PMR). The PMR values of cases and controls are 1.45 (0.87, 3.54) and 0.75 (0.59, 0.92), respectively.

**Figure 3:**
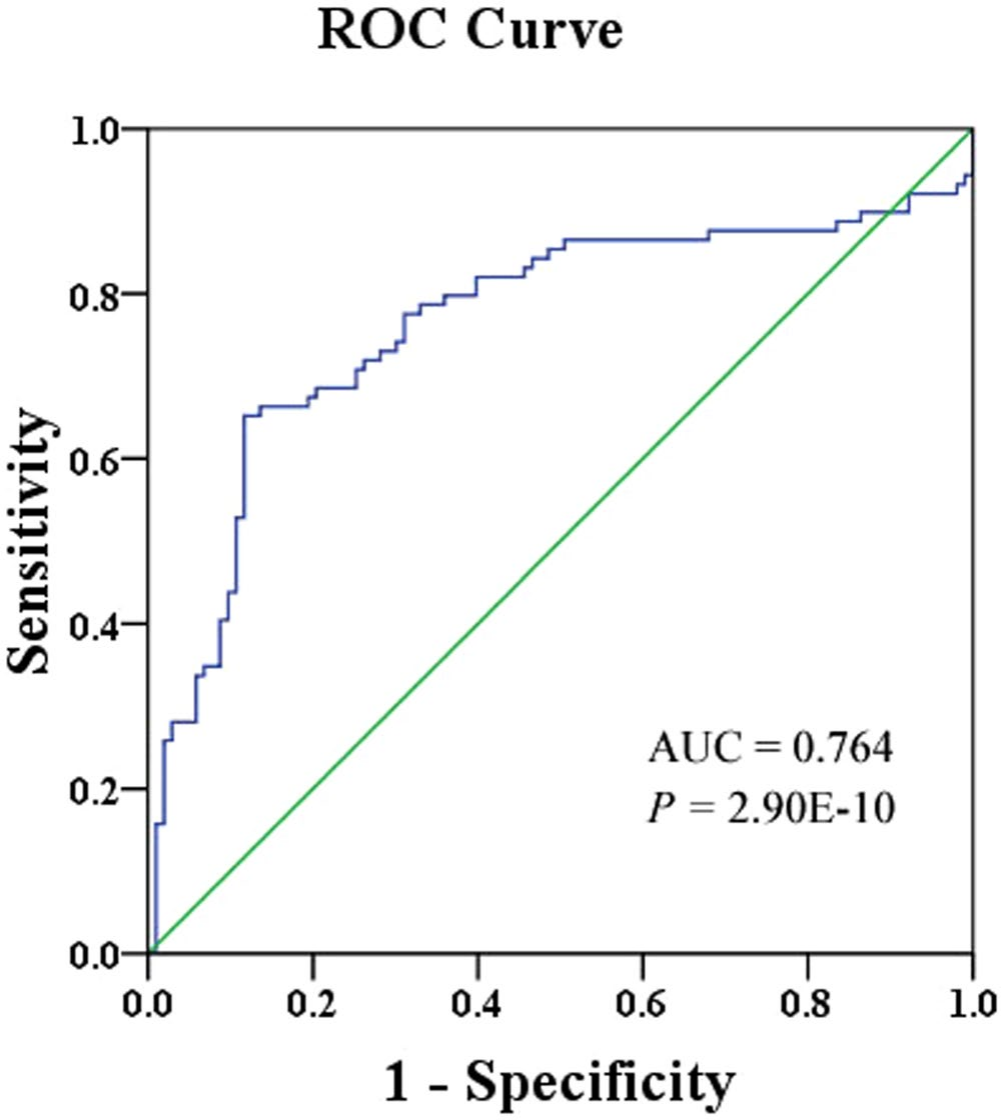
ROC curve for the diagnostic value of *UMOD* methylation ROC curve analysis of the *UMOD* gene hypermethylation in gout patients from healthy controls. ROC stands for receiver operating characteristic. AUC stands for the area under the curve. The AUC of *UMOD* methylation in gout was 0.764 (0.690, 0.836) with a sensitivity of 65.2% and a specificity of 88.3%.

A fragment located in CpG (cytosine-phosphate-guanine) island of *UMOD* (Chr16: 20,344,373-20,364,037), hg19) was selected for the methylation assay (Fig. 1A). DNA sequence analysis showed that the bisulphite conversion of the template DNA was successful (Fig. 1B). Capillary electrophoresis confirmed that the amplified fragment length was 73 bp (Fig. 1C). As shown in Fig. 2, *UMOD* hypermethylation was significantly associated with the risk of gout. *UMOD* methylation was elevated in the gout cases compared with the controls [median (interquartile range): 1.45 (0.87, 3.54) versus 0.75 (0.59, 0.92), *P* < 0.001]. Subsequently, we analyzed the diagnostic role of *UMOD* hypermethylation in peripheral blood, obtaining an AUC of 0.763 (*P* = 2.90E-10, Fig. 3). The ROC curve showed that *UMOD* methylation was a promising biomarker for gout (sensitivity = 65.2%, specificity of 88.3%).

In order to investigate the relationship between *UMOD* methylation and the pathogenesis of gout, the correlation tests were performed between *UMOD* methylation levels and clinical features in control samples. Significant inverse correlation was found with *UMOD* methylation level and UA (r = −0.208; *P* = 0.035, Table 2). However, there was no significant association between clinical features (age, ALT, AST, CRE, Glu, cholesterol, HDL, LDL, TG, WBC) and *UMOD* methylation (all *P* > 0.05, Table 2).

**Table 2:** Associations between *UMOD* methylation levels and clinical indexes features in normal controls.

Characteristics	r	*P* value*^a^
Age	−0.008	0.938
ALT	−0.151	0.127
AST	−0.043	0.667
CRE	−0.126	0.206
UA	−0.208	**0.035**
Glu	0.074	0.455
Cholesterol	0.001	0.988
HDL	0.088	0.374
LDL	0.041	0.681
TG	−0.185	0.062
WBC	−0.098	0.325

^*^The value in bold indicates statistical significance. ^a^Spearman test was applied. ALT: glutamic pyruvic transaminase; AST: glutamic oxalacetic transaminase; CRE: creatinine; UA: uric acid; Glu: blood glucose; HDL: high-density lipoprotein; LDL: low-density lipoprotein; TG: triglyceride; WBC: white blood cell.

**Figure 4:**
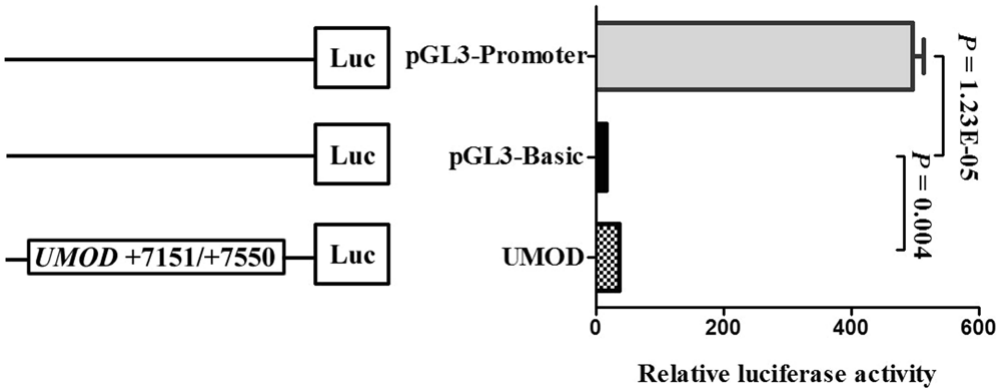
Dual-luciferase reporter assay in HEK-293T cell line. The pGL3 Basic and promoter vectors were used as negative and positive control in this study, respectively. Relative luciferase activity was performed in triplicates.

We performed a dual-luciferase reporter assay to check whether the *UMOD* CpG island region (+7151 bp to +7550 bp) was able to regulate gene expression. Our results showed that the transcriptional activity of recombinant pGL3-*UMOD* plasmid was higher compared with that of empty vector pGL3-basic (mean ± sd: 36.22 ± 2.15 versus 17.11 ± 0.16, fold change = 2, *P* = 0.004, Fig. 4).

## Discussion

In the present study, we reported for the first time that *UMOD* hypermethylation was significantly associated with the risk of gout in Chinese male patients. Moreover, the methylation levels of *UMOD* could be served as a predictive biomarker for the risk of gout.

DNA methylation has been studied in many metabolic diseases. *Prdx2* and *SCARA3* hypermethylation played an important role in the pathogenesis and progression of diabetes mellitus^31^. In diabetic ketoacidosis, *POMC* hypomethylation might make the patients’ condition worse^32^. Moreover, *AR* methylation was shown to be associated with hyperuricemia^33^. However, there were few articles between DNA methylation and gout. Previous studies showed that uromodulin (*UMOD*) played an important role in gout^34^. *UMOD* encoded the uromodulin glycoprotein. The mutations of *UMOD* led to uromodulin glycoprotein dysfunction and gout^35^.

As shown in the genecards website, *UMOD* expression level is able to be detected in the whole blood according to both the microarray and the RNAseq technologies. *UMOD* expression level is the highest in kidney, and uromodulin is the most abundant urine protein^36^. Decreased serum uromodulin is often correlated with the increase of serum inflammatory cytokines and the aggravation of diseases including kidney disease, hypertension and diabetes^11, 36–38^. In addition, the increase of serum uromodulin was a promising prognostic biomarker for recovery from acute kidney injury^39^. Besides, another kidney-specific gene, *Klotho* (*KL*) was reported to be much less expressed in peripheral blood cells compared in kidney^40^. *KL* hypermethylation in peripheral blood mononuclear cells was detected to be associated with the aggravation of chronic kidney disease^41^.

In the current study, elevated *UMOD* methylation in peripheral blood was shown to be associated with the risk of Gout, which is characterized by urate crystal-induced inflammation^42^. Since *UMOD* expression was often inversely associated with the levels of inflammatory cytokines in peripheral blood^11^, we speculate that elevated *UMOD* methylation in Gout might reduce the expression of *UMOD*, which triggers an immune response and leads to the risk of gout. In addition, our study couldn’t exclude the possibility that *UMOD* hypermethylation (and possibility of other genes) in peripheral blood cells could be secondary to increased circulating levels of uric acid (or of other molecules found to be increased in cases). Future study is warranted to investigate the correlation of *UMOD* methylation with *UMOD* expression in peripheral blood, kidney and other tissues.

In our study, a significantly higher serum UA level was found in gout patients than that in normal controls, and this finding might support that an elevated serum UA concentration was the main cause of gout^43^. But a significant inverse correlation was found between *UMOD* methylation level and serum UA level in controls. Due to the limited samples, we didn’t measure uromodulin levels in serum or urine in cases and controls in time. Therefore, we couldn’t test the correlation of *UMOD* expression and *UMOD* methylation in the samples. The relationship between *UMOD* methylation and the pathogenesis of gout needs further investigation.

Joint aspiration with synovial fluid analysis for monosodium urate crystals were the reference standard in early diagnosis of gout, however, rarely patients used this method in the early diagnosis of gout due to the risk of infection^44^. Our ROC curve analysis showed a moderate sensitivity of 65.2% and a high specificity of 88.3%. Moreover, increased levels of uric acid in blood is one of the clinical diagnostic criteria for gout^45, 46^. However, the blood uric acid index does not seem sensitive enough, patients with early-onset gout do not have a significant increase in uric acid levels^47^. And the detection rate of gout by using serum uric acid had a relatively low AUC of 0.61^48^. These findings suggested that *UMOD* methylation could be a diagnostic biomarker for gout. Dual-luciferase reporter system assay is a common tool to verify whether the cloned DNA fragment can play a regulation role in the expression of the luciferase reporter gene^49^. HK293T cell line was chosen for its easy culture and transfection. In the current study, pGL3-*UMOD* recombinant plasmid was constructed, and it was co-transfected into cells along with an internal control vector (pRL-SV40). Our results showed that the specific fragment (+7151 bp to +7550 bp) in *UMOD* CpG island region could induce a significantly higher expression of reporter gene than the control. Besides, as shown in the Supplementary Figure 1, other 400-bp inserts didn’t show obvious promoter activities, suggesting the *UMOD* fragment contained DNA elements with gene up-regulation. According to the TCGA dataset (https://genome-cancer.ucsc.edu/), there were five CpGs (cg03140788, cg06294373, cg21996068, cg09792189 and cg00376654) on the 400 bp fragment and three CpGs (cg06294373, cg21996068 and cg09792189) in the 73 bp fragment. Using the TCGA data, we found all the five CpGs were in positive correlation (r > 0.25, P < 0.001), suggesting that the selected CpGs might represent the neighbor CpG sites. In addition, UCSC Genome Browser website showed that the fragment was overlapped with several transcription factors binding sites, such as CTCF and ZNF143. We used P-Match method^50^ to predict TFBS in the selected fragment, there were Nkx2-5, c-Rel, NF-kappaB(P65), NF-kappaB in this fragment. Further study should be performed to explore the regulatory roles of CpG region in *UMOD* expression.

In conclusion, our study found that *UMOD* DNA hypermethylation in peripheral blood might be used to predict the risk of gout.

## Electronic supplementary material

Supplementary Information
