# Supplementary material for: Elevated UMOD methylation level in peripheral blood is associated with gout risk
Source: Sci Rep. 2017 Sep 11;7:11196. doi: 10.1038/s41598-017-11627-w (PMC5593964; doi:10.1038/s41598-017-11627-w)
Supplement: Supplementary file 1 — Supplementary Information [file 41598_2017_11627_MOESM1_ESM.doc]

**Elevated *UMOD* methylation level in peripheral blood is associated with gout risk**

Yong Yang^#^, Xiaoying Chen^#^, Haochang Hu^#^, Yuting Jiang, Hang Yu, Jie Dai, Yiyi Mao, Shiwei Duan^*^

Medical Genetics Center, School of Medicine, Ningbo University, Ningbo, Zhejiang 315211, China

#: YY , XC and HH are co-first authors of this work.

*: Correspondence should be addressed to Dr. Shiwei Duan ([duanshiwei@nbu.edu.cn](mailto:duanshiwei@nbu.edu.cn))

Figures

**
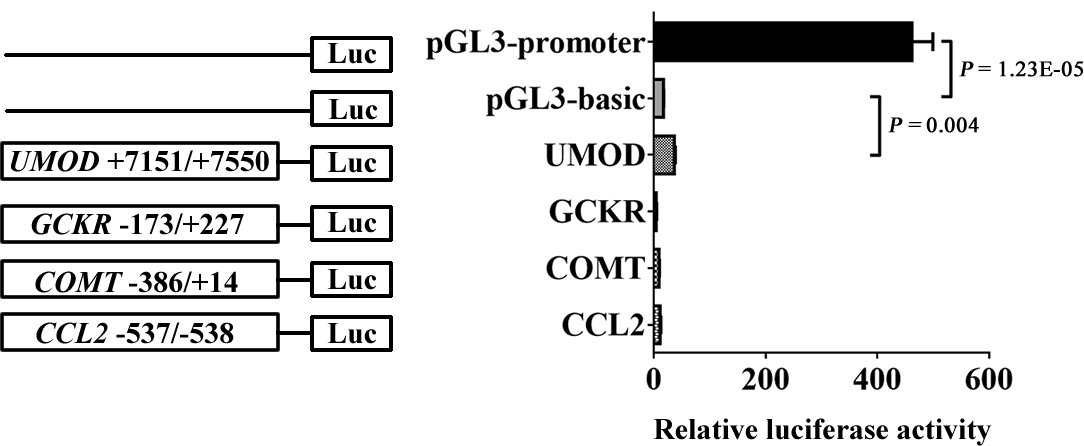
**

**Supplementary Figure 1:** Dual-luciferase reporter assay in HEK-293T cell line. The pGL3 Basic and promoter vectors were used as negative and positive controls in this study, respectively.
